# Supplementary material for: Patterns of Intron Gain and Loss in Fungi
Source: PLoS Biol. 2004 Nov 30;2(12):e422. doi: 10.1371/journal.pbio.0020422 (PMC532390; doi:10.1371/journal.pbio.0020422)
Supplement: Table S1 — Also available at http://genes.mit.edu/NielsenEtAl/. (4.3 MB ZIP). [file pbio.0020422.st001.zip › NielsenEtAl/html/362.html]

AN1555.1.NCU09324.1.MG09962.1.FG01272.1


```
 CLUSTAL W (1.82) Multiple Sequence Alignments - Introns Inserted


Sequence 1: NCU09324.1	1195 aa
Sequence 2: MG09962.1	1208 aa
Sequence 3: FG01272.1	1222 aa
Sequence 4: AN1555.1	1184 aa
Alignment Length: 1268 aa
Number Identitical Residues: 636 aa
Alignment Score (without introns) 30814


MG09962.1 	MSLPERPGAKASYEQRNSYRKSPSRRNRPNDIEASGYYPVT-GGQHQRGPSVNSFAETIR
NCU09324.1	-MAGERP------------------------------------SQQQRVPSISSFPETLP
FG01272.1 	MSLPERPGASQAYNQRSVYRNSPSRRSRPVDIETGGYHAVDNQASHQRGKSGSSFAESIG
AN1555.1  	MSLPQRPGKTSPRREETSAFREP--------------------SRRRRRESDSLSNNDPT
          	    :**. . .  . .    ..                    . ::*  * .   :   

MG09962.1 	SPNSNIESAPLSPSA---EQIEHGSDQPFQRKRSLIRPERNRIDRDHPNYHYRKHAAKMN
NCU09324.1	SPNPNVERDPLAPTP---EPAHPSGIP--QRKRSLIRPERNRIGKDHPNYHYRKHAANMN
FG01272.1 	-INSNTESMPLSPTSPDGQSRHAGPEQPISRKRSLIRPERNRIDRDHRNYHYHKHAAHMN
AN1555.1  	SPRHHRHHRSHSSRHQHDIDEERAEEGGIRRKRSLVKPERGRMDPSHPNYLYRQKTQNMP
          	: . : .  . :.    .   . .      *****::***.*:. .* ** *:::: :* 

MG09962.1 	TLPSSTGNDPVLEDVSGATESGPPSGSNSASGSGVREENIPRKSRKASGRETVAEKSDNT
NCU09324.1	TLPSSTGHDPIYEDLEGATDD--VSGTGSRNDDDVSEESPPRRKHSTKMQVIETEKSGDE
FG01272.1 	VLPSSTGNDPIYEDFEASTER-----TNSNYNEGGSDGS-PRQRRTVSG---DHEKSAAV
AN1555.1  	TYNPMTGNEPLIHEEGEAETN-----STPSMDSKRKDALYGAHGNVNKPMERVPTRHRSK
          	.  . **::*: .:   :        : .  ..   :     : .  .       :    

MG09962.1 	RRR----VSTRNSKIVKEGKRKEKIP----EQLRPPSAWNVYCAVITFWSPDFIMKCCGM
NCU09324.1	RRRRRKSDTTKHGKIVKASKGKREKS----GGLPTPSFWNIYCGFVTFWCPGFVLKCFGM
FG01272.1 	ASTLPTPDRTKSGKIKRKSRRHSKPPKRIEEQLRPPTFWNVYCAIVTFWAPGFIMKCCGM
AN1555.1  	KRK----GSRKISKREAAAEKRRRKA---MEQVRPPSLWTTYCSVITFWAPDFVLKCFGM
          	          : .*    .. : . .      : .*: *. **..:***.*.*::** **

MG09962.1 	PAKAQRRAWREKIGLISLILIIMGVVGFLTFGFNQAVCGGPVLRLHINSVDRSYMIFHGT
NCU09324.1	PEMAQQRAWREKMGLISIILLIMGFVGFITFGFTQVVCGKPPLRLRINEVGSGYMIFHGS
FG01272.1 	PTRAQQRAWREKMGLISIILIIMAIVGFLTFGFTATVCGAPQERLRVNKVDGGNMIFHGV
AN1555.1  	PQKAQRSAWREKIGLISIILMIAAFVGFLTFGFTATVCGTPPTRLKINEIGSGYMIFHGQ
          	*  **: *****:****:**:* ..***:****. .*** *  **::*.:. . ***** 

MG09962.1 	AYNLDGSHHPVAEGIPKRLDGTGANVVYDLPEGYGGTDGSFMFQNVNGKCKGLITKAPNS
NCU09324.1	AYDLTKSHHPPAEGIPRRPDGLGANVIYDLPQHYGGQDGSFLFQNVNGKCKGLITKQENS
FG01272.1 	AYDLSESHHPPAQGMPLRSDGLGPNVLYDLPEKNAGKDGSFLFQNVNGRCKGLITLAKGS
AN1555.1  	AYDLTKSTHPAAAGIPDMT-----NVLYDLPHKYGGQDGSFFFQEVNGACKGLITRTENS
          	**:*  * ** * *:*        **:****.  .* ****:**:*** ******   .*

MG09962.1 	DVPSE-GDNLAWYFPCHARNQDGSSQPNMTYPYYFGYACHTTPLARSTFYTQLDKSADVY
NCU09324.1	DVPSDKSGNLAWYFPCNTFNQDGSSKPNTTIPYYLGYACHTTANARDSFYLGLKSSADVY
FG01272.1 	DVPTNDDGDLAWYFPCKTFNQDGSSKVNTTTPYYSGYGCHTTLDSRNAFYLDLKGAADVY
AN1555.1  	DIPTNSNGDLAWYFPCHAFNQDGSSEPNTTVSYYNGWACHTSGSARKSFYS-LKNSGDVY
          	*:*::...:*******:: ******: * * .** *:.***:  :*.:**  *. :.***

MG09962.1 	FTWADIRNNSRNLFVYSGNVLDLDLLFWFNRDQVNIPRRFEELRDKNNAANRAIRGRDAT
NCU09324.1	FTWDDIKNSSRNLVVYSGHVLDLDLLHWFNDTQVTYPARFKELRDKNTAGNQAIRGRDIT
FG01272.1 	FTWDDIKNKSRNLVVYSGNVLDLDLLKWFNSTQVDIPERFKELRDKNSAVNKAIRGRDVT
AN1555.1  	FTWEDTKNTSRKLAVYSGNVLDLNLLNWFDDTQVNYPTKFKDLRD-----NDDIRGVDLT
          	*** * :*.**:* ****:****:** **:  **  * :*::***     *  *** * *

MG09962.1 	RTFMASGDRQIAECFEDIIKVGTVDTDTVGCIAAKVVLYVSLALILSVVGARFTLALIFQ
NCU09324.1	HAFQSSKDKQIAECFEEIIKVGSVDTETVGCIASKVVLYVSLVLILAVVLARFVLALIFQ
FG01272.1 	RMFQSSSDKKYAQCFEEIIKVGSVDTETIGCIASKIVLYCALALILSVVGVRFFLAIIFQ
AN1555.1  	YYFQTGEDKQIGKCLSQIIKVGSIDTDTVGCIASQVVLYVSLIFILSIVIVKFAFALLFQ
          	  * :. *:: .:*:.:*****::**:*:****:::*** :* :**::* .:* :*::**

MG09962.1 	WFISKNYAADKTSQ-TSDKRKRNKQIEDWSEDIYRAPPRMPGDVGSSVAGASSSDHTSKR
NCU09324.1	WFISKTYAAAKTSQ-TSDQRKRNRQIEDWTEDIYRAPPRLPGEVGSSVAGSS--DRQSKR
FG01272.1 	WFICRKYAPTKTSQ-SSDRRKRNKQIEDWSEDIYRAPIRLPGDVGSTVYGSS--DRSSKR
AN1555.1  	WFLAPRFAAQKTSMGAVDSKARNQQIEDWSNDIYRPGPRLADPVPG--------DRMSKR
          	**:.  :*. *** .: * : **:*****::****.  *:.. * .        *: ***

MG09962.1 	S-SFLPTTSRFSTVYG-AERSARNKSMPTTMASQASGGYMGPSST-----AYRETNE---
NCU09324.1	SSAFLPTHSRFSTVYG-NERGNRKPGLPTTMASQNAAGQLLHPGT-----IYGQGNE---
FG01272.1 	A-SFLPTTSRFSSVGGPDIRSQGGRRMPTTMASQSTSNQLLTPNS-----MFKQGND---
AN1555.1  	A-SFLPTTSRFSSPYT-VSNGGKQKPQWVTMASQNSTTRLVPPASGTTPSIYRQSHNGLG
          	: :**** ****:      ..       .***** :   :  . :.::.: : : ::. .

MG09962.1 	-------------SRTSFLKSDPYATNTAPIEGPGPSGFIHDSVVPQPPSDWMPFGFPLA
NCU09324.1	-------------SRSSFLKSDAYGSSSSPADGPGPAGFIHEAVVPQPPSDWMPFGFPLA
FG01272.1 	-------------SRASFLRTDPYS--STPTDGPGPAGFIHDAVVPQPPSDWMPFGFPLA
AN1555.1  	NVSVDNSRLNPSASRTSLVQDSRYSTVIPDSEGIGSAGYVHELVVPQPPPDWQPYGFPLA
          	. : ..:  ..::**:*::: . *.:  .  :* *.:*::*: ******.** *:*****

MG09962.1 	HTICLVTAYSEGELGLRTTLDSVAMTDYPNSHKVILVICDGIIKGKGESKSTPEYVLDMM
NCU09324.1	HTICLVTAYSEGEMGVRTTLDSIAMTDYPNSHKVILVICDGIIKGHGEEHSTPDIILGMM
FG01272.1 	HTMCLVTAYSEGEEGIRTTLDSIATTDYPNSHKVIVVICDGIIKGQGETMSTPDVCLGML
AN1555.1  	HAMCLVTCYSEGEEGIRTTLDSIALTDYPNSHKSIVVICDGIIKGKGEEFSTPDIVLRMM
          	*::****.***** *:******:* ******** *:*********:**  ***:  * *:

MG09962.1 	KDHTIPVEDVEAFSYVAVASGSKRHNMAKIYAGFYDYGTQSNIPLDKQQRVPMMVVVKCG
NCU09324.1	KDHTIHPDDVEPFSYVAVATGSKRHNMAKVYTGFYDYGTNSAIPLEKQQRVPMMMVVKCG
FG01272.1 	KDHSIPPDMVEPFSYVAVASGSKRHNMAKIYCGFYDYGKNSRIPADRQQRVPMMVVVKCG
AN1555.1  	RDPIIPPEEVEAFSYVAVATGSKRHNMAKVYAGFYDYGEHSIIPVEKQQRVPMMIIVKCG
          	:*  *  : **.*******:*********:* ****** :* ** ::*******::****

MG09962.1 	TPDEMVKSKPGNRGKRDSQIILMSFLQKVMFDERMTELEYEMFNGLWKVTGISPDFYEIV
NCU09324.1	TPAEASKSKPGNRGKRDSQIILMSFLQKVMFDERMTELEYEMFNGLWKITGISPDFYEIV
FG01272.1 	TPDEASKSKPGNRGKRDSQIILMSFLQKVMFDERMTELEYEMFNGLWKVTGISPDYYEII
AN1555.1  	TPAEATAAKPGNRGKRDSQIILMSFLQKVMFDERMTELEYEMFNGLLHVTGIPPDFYEVV
          	** *   :************************************** ::***.**:**::

MG09962.1 	LMVDADTKVFPDSLTHMISAMVKDPEIMGLCGETKIANKRDSWVSAIQVFE~YFISHHLA
NCU09324.1	LMVDADTKVFPDSLTHMISAMVKDPEIMGLCGETKIANKRASWVSAIQVFE~YFVSHHLA
FG01272.1 	LMVDADTKVFPDSLTHMISAMVKDPEIMGLCGETKIANKRDSWVTAIQVFE~YFVSHHLA
AN1555.1  	LMVDADTKVFPDSLTHMISAMVKDPEVMGLCGETKIANKTDSWVTMIQVFE2YFVSHHQS
          	**************************:************  ***: ***** **:*** :

MG09962.1 	KSFESVFGGVTCLPGCFCMYRIKAPKGAQNYWVPILANPDVVEHYSENVVDTLHKKNLLL
NCU09324.1	KAFESVFGGVTCLPGCFCMYRIKAPKGAQNYWVPILANPDVVEHYSENVVDTLHKKNLLL
FG01272.1 	KSFESVFGGVTCLPGCFCMYRIKAPKGGHNYWVPILANPDIVEHYSENVVETLHEKNLYL
AN1555.1  	KAFESVFGGVTCLPGCFSMYRIKAPKGGQNYWVPILANPDIVEHYSENVVDTLHKKNLLL
          	*:***************.*********.:***********:*********:***:*** *

MG09962.1 	LGEDRYLTTLMLRTFPKRKQVFVPQAVCKTTVPDSFMVLLSQRRRWINSTIHNLMELVLV
NCU09324.1	LGEDRYLSTLMLRTFPKRKQVFVPQAVCKTTVPDEFMVLLSQRRRWINSTIHNLMELVLV
FG01272.1 	LGEDRFLTTLMLRTFPKRKQVFIPQAVCKTTVPDEFMVLLSQRRRWINSTIHNLMELVLV
AN1555.1  	LGEDRYLSTLMLRTFPKRKQIFVPQAVCKTVVPDKFMVLLSQRRRWINSTVHNLMELVLV
          	*****:*:************:*:*******.***.***************:*********

MG09962.1 	RDLCGTFCFSMQFVIFIELIGTLVLPAAIAFTFYVV1IISIINQPPQIIPLVLLGLILGL
NCU09324.1	RDLCGTFCFSMQFIVGIELIGTLVLPAAIAFTFYVV1IISIINSPPQIIPLVLLGLILGL
FG01272.1 	RDLCGTFCFSMQFIIFVELVGTLVLPAAIAFTFYVV1ITSIINSPPQIIPLVLLGLILGL
AN1555.1  	RDLCGTFCFSMQFVIFVELVGTVVLPAAISFTIYVV~VSSIIKQPVQIIPLVLLALILGL
          	*************:: :**:**:******:**:*** : ***:.* ********.*****

MG09962.1 	PAILIIITAHSWSYVLWMLIYLLSLPVWNFVLPAYAFWKFDDFSWGDTRKTAGEKTKKAG
NCU09324.1	PAILVVVTAHSWSYIIWMFIYLLSLPVWNFVLPTYAFWKFDDFSWGDTRKTAGEKSSKGG
FG01272.1 	PGLLVIITAHSWSYIVWMLIYLLALPIWNFVLPTYAFWKFDDFSWGETRKTAGEKTKKAG
AN1555.1  	PGVLVVVTAHRLVYVLWMLVYLISLPIWNFVLPTYAYWKFDDFSWGDTRKTAGEKDKGHE
          	*.:*:::***   *::**::**::**:******:**:*********:******** .   

MG09962.1 	-IEYEGEFDSSKITMKRWAEFERDRRARQSQYWGSREN0---------------------
NCU09324.1	HGEAEGEFDSSMITMKRWAEFERDRRVRSTYWAGSRDN~VISGVGGSNGWGSSQPRGHEQ
FG01272.1 	-LEYEGEFDSSKITMKRWAEFERDKRSRSG-YWGSREN~VIGG-------GGQTWTSPPG
AN1555.1  	--DGEGEFDSSKITMKRWRDFEKGMSMLTVLHDARTAN~---------------------
          	  : ******* ****** :**:.         .   *                      

MG09962.1 	-QQYDEYYSDA
NCU09324.1	GRHFDDYFSDA
FG01272.1 	HQYNEEYYSDA
AN1555.1  	----DIY----
          	    : *
```
